# Improving Adherence to the Driver and Vehicle Licensing Agency (DVLA) Guidance on Documented Driving Advice for Psychiatric Patients Managed by a Home Treatment Team (HTT): A Two-Cycle Quality Improvement Project

**DOI:** 10.1192/bjo.2026.11160

**Published:** 2026-06-30

**Authors:** Chinenye Ezemagu-Onedi, Ella Fisher

**Affiliations:** Royal Devon Hospital (Northern Division), Barnstaple, United Kingdom

## Abstract

**Aims:**

Psychiatric conditions can impair fitness to drive, and clinicians have a professional duty to provide and document appropriate advice in accordance with DVLA guidance. This quality improvement project (QIP) aimed to evaluate adherence to DVLA guidelines regarding the provision of documented driving advice to patients with psychiatric conditions under the Home Treatment Team (HTT), firstly as a baseline measurement, and after meaningful intervention. Identifying disparities in compliance across different diagnosis, sex and age, to enhance patient’s safety and clinical practice.

**Methods:**

An initial retrospective review of HTT electronic patient records was conducted over three months (June–August 2024) for a total of 86 patients. Variables collected included primary psychiatric diagnosis, age, sex and whether DVLA driving advice was documented as given. Standards were based on DVLA guidance “Assessing fitness to drive: a guide for medical professionals”. Following baseline measurement, a multi-strategy intervention package was implemented: (1) Multi-Disciplinary Team (MDT) checklist prompt to ensure driving status and advice were considered and recorded during the MDT, (2) Patient information leaflet summarising DVLA advice, (3) Presentation of initial findings at Learning-From-Excellent meeting, and (4) Staff education leaflet and teaching highlighting the clinical and medico-legal importance of driving advice. A second retrospective audit cycle was completed using identical methodology over four months (January–April 2025) for a total of 93 patients, again evaluating documentation of DVLA guidance being given.

**Results:**

Baseline documentation of driving advice was present in 25/86 records (29.1%), with 61/86 (70.9%) illustrating no documented driving advice. Following our intervention, documentation improved to 76/93 records (81.7%), with 17/93 (18.3%) lacking documentation, showing an absolute improvement of +52.6%.

In the second cycle (n=93), 59 records related to female patients and 34 to male patients. Driving advice was applicable in 54/93 cases (36 female; 18 male). Where applicable, documented advice was provided in 29/36 female cases (80.6%) and 14/18 male cases (77.8%), demonstrating comparable adherence across sexes. Among those given advice, the most common age group was 40–60 years for both females (12/29; 41.4%) and males (7/14; 50.0%).

**Conclusion:**

This two-cycle QIP demonstrated substantial improvement in adherence to DVLA guidance documentation within HTT, increasing from 29% to 82% following low-cost meaningful interventions embedded into MDT workflow and staff education. Post-intervention data revealed similar compliance across sexes, with most documented advice delivered to patients aged 40–60 years. Future work should assess the quality of driving advice given and strategies to support consistent practice.

